# Post-traumatic Stress Disorder Among Healthcare Workers Diagnosed With COVID-19 in Jeddah, Kingdom of Saudi Arabia, 2020 to 2021

**DOI:** 10.7759/cureus.17371

**Published:** 2021-08-22

**Authors:** Amjed S Alshehri, ‏Amal H Alghamdi

**Affiliations:** 1 Directorate of Health Affairs for Public Health Division, Ministry of Health - Kingdom of Saudi Arabia, Jeddah, SAU; 2 The Saudi Joint Program for Preventive Medicine, Ministry of Health - Kingdom of Saudi Arabia, Jeddah, SAU

**Keywords:** ptsd, healthcare workers, covid 19, post traumatic stress disorder, health care workers, infectious diseases, mental health

## Abstract

Background

Post-traumatic stress disorder (PTSD) is a global problem. According to its definition, it is a disorder that occurs with some people who have undergone or witnessed a shocking, terrifying, or hazardous event, and the coronavirus disease (COVID-19) with its consequent threats and fear meets the definition of a traumatic event. The main aim of this study is to determine PTSD in healthcare workers (HCWs) who survived COVID-19 in Jeddah, Saudi Arabia.

Subjects and methods

Through an analytical cross-sectional study, HCWs working in Jeddah city with a minimum of seven days since their first positive COVID-19 result were included in this study. They were screened using the 'PTSD checklist for The Diagnostic and Statistical Manual of Mental Disorders (DSM-5)' (PCL-5), which is a 20-item self-report measure that assesses the presence and severity of PTSD symptoms.

Results

Out of all respondents (n=404), there was slight dominance of females (54.0%) over males (46.0%), and an almost equal distribution of Saudis (51.2%) and non-Saudis (48.8%); their mean age accounted for 36.9±8.7 years. PTSD was detected in 14.9%; the prevalence was significantly higher in those who had chronic diseases (23.7%), especially diabetics (30.8%) and obesity (41.2%), HCWs whose job necessitates exposure to positive cases (18.8%), and those who were isolated in hospitals while being ill. All the above values were statistically significant (p<0.05).

Conclusion and recommendations

The prevalence of PTSD in the HCWs who survived COVID-19 came within the range of that in HCWs who were dealing with cases of COVID-19 without being affected. Efforts should be made to alleviate stress in HCWs during their work in pandemics.

## Introduction

Coronaviruses are a large family of viruses that can cause infection in both animals and humans. In humans, several coronaviruses are known to cause respiratory infections ranging from the common cold to severe diseases like the Middle East respiratory syndrome (MERS) and the severe acute respiratory syndrome (SARS). The recently detected coronavirus, SARS-CoV-2, causes coronavirus disease (COVID-19). COVID-19 was unknown before the outbreak started in Wuhan, China, in December 2019, and by early 2020, It was affecting many countries globally and consequently declared as a pandemic [1]. As a result of its fast spread, the COVID-19 pandemic did not only raise public health concerns but also caused significant psychological consequences.

Post-traumatic stress disorder (PTSD) was first introduced as a diagnosis in 1980 [2]. PTSD is a global problem associated with persistent disability and comorbidity for many people. It is a disorder that may occur with people who have undergone or witnessed a shocking, terrifying, or hazardous event. People go through various responses after trauma; however, most people recover from initial symptoms like ﻿distressful memories and flashbacks, avoidance, and hypervigilance spontaneously. Those who do not recover spontaneously and continue to experience the symptoms may be diagnosed with PTSD. People with PTSD could experience frightening or stressful feelings, even when they are not in danger anymore [3]. Critical events include death, life-threatening injury, or a crisis with a need for rescue or emergency, resulting in stress-related reactions and the development of PTSD [4]. According to the definition, getting infected with COVID-19, along with the collective and personal threats and fear that it could cause, meets the definition of a traumatic event. Exposure to such traumatic events can lead to acute stress disorder and finally PTSD if symptoms persist. Major natural disasters and pandemics are usually related to remarkable increases in mental health disorders among healthcare workers (HCWs) [5].

It is essential to evaluate the mental health of HCWs diagnosed with COVID-19, who are at high risk of developing stress reactions due to the nature of their work. The current study aimed at determining the prevalence and determinants of PTSD among HCWs diagnosed with COVID-19 in Jeddah during the COVID-19 pandemic.

## Materials and methods

Through an analytical cross-sectional study design, HCWs with a minimum of seven days since their first positive COVID-19 result were included in the study. The HCWs were screened using the 'PTSD Checklist for The Diagnostic and Statistical Manual of Mental Disorders (DSM-5)' (PCL-5), which is a 20-item self-report measure that assesses the presence and severity of PTSD symptoms. Translation and validation of the questionnaire to Arabic had been done in a previous study [6]. There was another validated self-administered questionnaire to identify the predictive factors of PTSD. Participants of the study signed informed consent and submitted the PCL-5 scale online. Ethical approval was ensured from the local IRB in Jeddah health affairs according to King Abdulaziz City of Science and Technology (Saudi Arabia) Good Clinical Research Practice (GCP) regulations (approval A01003).

The data were collected, coded, and analyzed using IBM SPSS Statistics for Windows, Version 21.0, released 2012 (IBM Corp, Armonk, NY). The continuous variables are presented as mean and standard deviation, while categorical variables are presented as frequency distribution and percentages. A Chi-square test was used to identify any association between the outcome and the independent variables. Binary logistic regression was done to identify predictors of PTSD. A 95% CI was adopted throughout the study; a p-value of less than 0.05 was considered as a level of significance. Quality control was done at the stages of coding and data entry.

## Results

According to the study design, 404 HCWs who had been diagnosed with COVID-19 were included in the study. There was slight dominance of females (54.0%) over males (46.0%), and almost equal distribution of Saudis (51.2%) and non-Saudis (48.8%). The mean age of the participants accounted for 36.9±8.7 years, about one-half of them aged 30-<40 years (46.8%) and one-third (33.2%) aged 40 years or older. Most of the participants had either bachelor’s qualification (51.2%) or a postgraduate degree (20.0%). Two-thirds are married (63.1%) and collectively 6.9% were either divorced or widowed. Accordingly, 60.6% reported that they had children and the majority (73.5%) are living in families. Only one quarter (25.5%) declared that they have a monthly income of less than 5,000 SR (1,300 $), while 5.2% have a monthly income of more than 30,000 SR (8,000 $) (Table 1).

**Table 1:** Socio-demographic characteristics of the study group (n=404).

Characteristics	No.	Percentage
Gender:		
Male	186	46.0
Female	218	54.0
Nationality:		
Saudi	207	51.2
Non-Saudi	197	48.8
Age categories:		
<30 years	81	20.0
30-<40 years	189	46.8
≥40 years	134	33.2
Education level:		
Postgraduate	81	20.0
Bachelor	207	51.2
Diploma	116	28.7
Marital status:		
Married	255	63.1
Single	121	30.0
Divorced	23	5.7
Widowed	5	1.2
Have children:		
Yes	245	60.6
No	159	39.4
Live with whom:		
Alone	59	14.6
Family	297	73.5
Friends	48	11.9
Household income:		
<5000 SR	103	25.5
5000-10000 SR	145	35.9
11000-20000 SR	102	25.2
21000-30000 SR	33	8.2
>30000 SR	21	5.2

**Figure 1:**
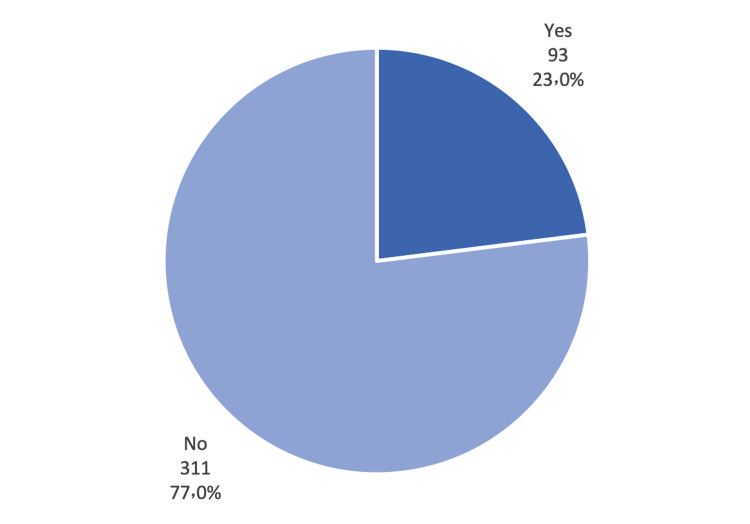
Chronic diseases reported by the respondents.

**Figure 2:**
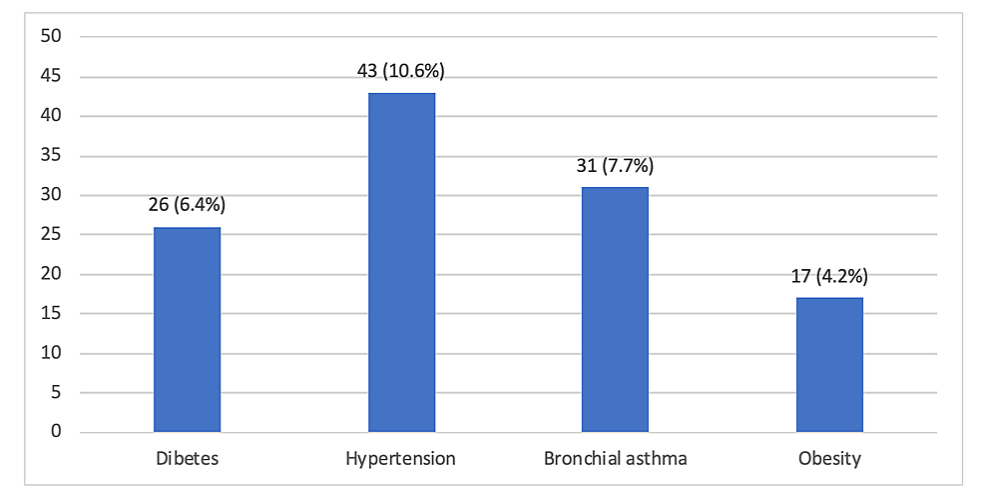
Types of chronic diseases reported by the respondents.

**Figure 3:**
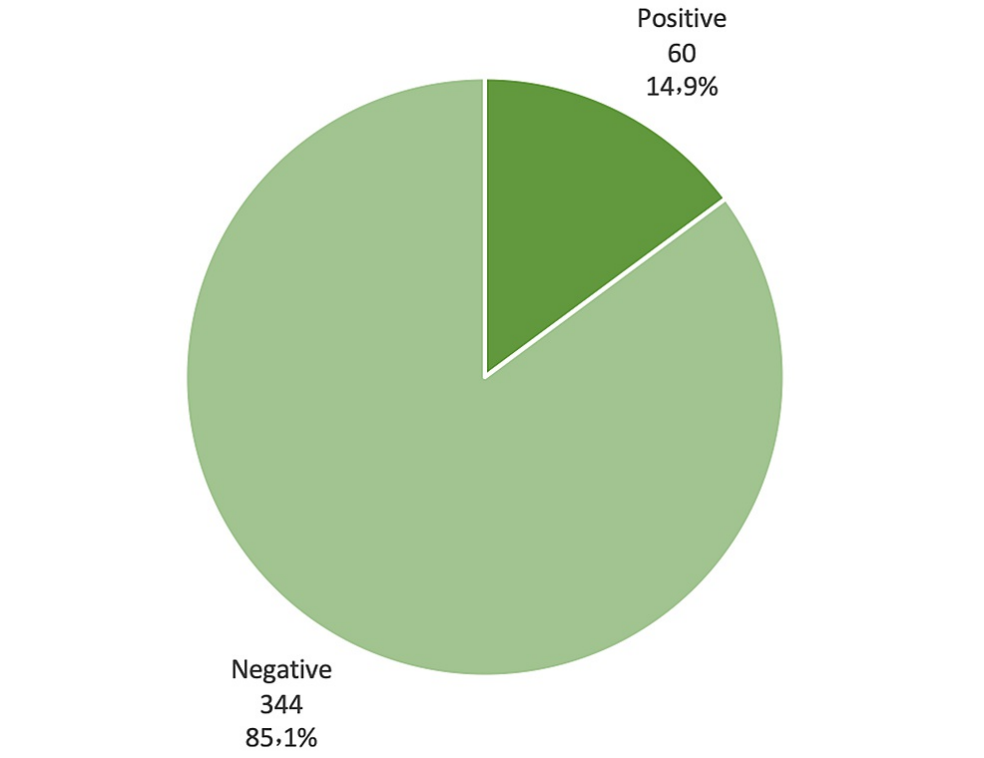
Classification of the respondents based on the overall PTSD diagnostic score.

Clinically, almost one-quarter of the participants (23.0%) indicated that they suffer from chronic diseases (Figure 1). The most commonly reported chronic disease was hypertension (10.6%) and bronchial asthma (7.7%), followed by diabetes mellitus (6.4%) and obesity (4.2%) (Figure 2). The overall score of the PTSD scale, with a cut-off level of "31", revealed that 60 out of 404 participants (14.9%) were positive for PTSD (Figure 3).

Table 2 shows the variation in the frequency of PTSD according to the demographic characteristics of the participants. Notably, it was observed that there was a stepwise consistent increase in the frequency of PTSD with increasing age. It ranged between 7.4% in young participants aged <30 years and 14.8% in those aged 30-<40 years, up to 19.4% in those aged 40 years or older, with a p-value of 0.057. The frequency of PTSD was significantly lower among non-smokers (12.3%) when compared to either smokers (20%) or ex-smokers (27.3%) (p<0.05).

**Table 2:** PTSD classification according to demographic characteristics of the respondents. * Based on Chi Square

	PTSD classification								
Variables	Positive		Negative						
	No	%		No	%		X^2^		P*
Gender:							0.033		0.861
Male	27	14.5%		159	85.5%				
Female	33	15.1%		185	84.9%				
Nationality:							0.399		0.527
Saudi	33	15.9%		174	84.1%				
Non-Saudi	27	13.7%		170	86.3%				
Age categories:							5.745		0.057
<30 years	6	7.4%		75	92.6%				
30-<40 years	28	14.8%		161	85.2%				
≥40 years	26	19.4%		108	80.6%				
Education level:							0.743		0.690
Postgraduate	11	13.6%		70	86.4%				
Bachelor	29	14.0%		178	86.0%				
Diploma	20	17.2%		96	82.8%				
Marital status:							3.816		0.148
Married	42	16.5%		213	83.5%				
Single	12	9.9%		109	90.1%				
Divorced/widowed	6	21.4%		22	78.6%				
Have children:							1.071		0.301
Yes	40	16.3%		205	83.7%				
No	20	12.6%		139	87.4%				
Live with whom:							1.843		0.398
Alone	9	15.3%		50	84.7%				
Family	47	15.8%		250	84.2%				
Friends	4	8.3%		44	91.7%				
Smoking status:							6.040		0.049
Smoker	18	20.0%		72	80.0%				
Non-Smoker	36	12.3%		256	87.7%				
Ex-smoker	6	27.3%		16	72.7%				
Household income:							4.606		0.330
<5000 SR	9	8.7%		94	91.3%				
5000-10000 SR	23	15.9%		122	84.1%				
11000-20000 SR	19	18.6%		83	81.4%				
21000-30000 SR	6	18.2%		27	81.8%				
>30000 SR	3	14.3%		18	85.7%				

In the same context, Table 3 shows that only contact with positive cases, as a part of the current position necessitating contact with COVID-19 positive cases, showed a statistically significant (p<0.05) difference between participants stating yes (18.8%) and no (11.3%), with an OD of 1.808 (95% CI; 1.034-3.160). On the other side, although the frequency of PTSD was higher among pharmacists (23.5%), there was a difference among those who had work experience between 5-<10 years (17.2%), those who are working 9-12 hours daily (16.1%), and those who are working in night shifts (25.0%). Nevertheless, these differences are not statistically significant (p>0.05). PTSD was significantly higher among those who had been isolated at hospitals (28.9%) as compared to those isolated at home (13.1%) or hotel quarantine (12.2%) p<0.05. The frequency was significantly higher among those who reported that they had chronic diseases (23.7%) p<0.05, with an OD of 2.226 (95% CI; 1.238-4.001). Specifically, the frequency was significantly much higher among diabetics (30.8%), with an OD of 2.786 (95% CI; 1.153-6.736) and obese HCWs (41.2%), with an OR of 4.411 (95% CI; 1.609-12.093) (Table 4).

**Table 3:** PTSD classification according to job characteristics of the respondents. * Based on Chi Square

	PTSD classification								
Variables	Positive		Negative						
	No	%		No	%		X^2^		P*
Job:							6.374		0.272
Physician	15	17.4%		71	82.6%				
Nurse	17	14.3%		102	85.7%				
Allied health personnel	16	18.8%		69	81.2%				
Pharmacist	4	23.5%		13	76.5%				
Dentist	0	0.0%		8	100.0%				
Others	8	9.0%		81	91.0%				
Years of experience:							2.906		0.234
<5 years	8	9.2%		79	90.8%				
5-<10 years	20	17.2%		96	82.8%				
10+ years	32	15.9%		169	84.1%				
Current position necessitate contact with positive cases:							4.397		0.036
Yes	36	18.8%		156	81.3%				
No	24	11.3%		188	88.7%				
Daily working hours:							0.567		0.904
<8 hours	2	12.5%		14	87.5%				
8 hours	31	14.8%		179	85.2%				
9-12 hours	23	16.1%		120	83.9%				
>12 hours	4	11.4%		31	88.6%				
Type of shifts:							1.011		0.799
Morning shift	28	14.5%		165	85.5%				
Evening shift	3	15.0%		17	85.0%				
Night shift	3	25.0%		9	75.0%				
Mixed	26	14.5%		153	85.5%				

**Table 4:** PTSD classification according to relevant clinical aspects of the respondents. * Based on Chi Square

	PTSD classification								
Clinical aspects	Positive		Negative						
	No	%		No	%		X^2^		P*
Fully recovered from COVID-19:							0.915		0.339
Yes	55	14.4%		326	85.6%				
No	5	21.7%		18	78.3%				
Place of isolation:							7.896		0.019
Home isolation	45	13.1%		298	86.9%				
Hospital admission	13	28.9%		32	71.1%				
Hotel quarantine	2	12.5%		14	87.5%				
Suffering from chronic diseases:							7.406		0.007
Yes	22	23.7%		71	76.3%				
No	38	12.2%		273	87.8%				
Diabetic							5.568		0.018
Yes	8	30.8%		18	69.2%				
No	52	13.8%		326	86.2%				
Hypertensive							0.536		0.464
Yes	8	18.6%		35	81.4%				
No	52	14.4%		309	85.6%				
Asthmatic							3.186		0.074
Yes	8	25.8%		23	74.2%				
No	52	13.9%		321	86.1%				
Obese							9.725		0.007
Yes	7	41.2%		10	58.8%				
No	53	13.7%		334	86.3%				

The regression model in Table 5 displays that the statistically significant predictor of consequent PTSD is having a chronic disease (p<0.05); with an OD of 2.046 (95% CI; 1.107-3.781), which means having a chronic disease duplicate the risk of having consequent PTSD after COVID-19 infection.

**Table 5:** Factors predicting PTSD in the respondents.

	B	S.E.	Wald	df	Sig.	Exp(B)	95% CI	
							Lower	Upper
Hospital isolation	.663	.389	2.907	1	.088	1.942	.905	4.163
Current position necessitate contact with positive cases.	.535	.296	3.271	1	.071	1.707	.956	3.047
Has chronic disease	.716	.314	5.211	1	.022	2.046	1.107	3.781
Constant	-2.325-	.244	90.527	1	.000	.098		

## Discussion

Since the WHO declared the rapid spread of COVID-19 as a pandemic, there has been a dramatic increase in the prevalence of mental and psychological problems in the general population worldwide [7]. Due to variation in potentiating risk factors of psychological distress, there are differences in the prevalence of distress in particular subgroups [8-10]. For example, HCWs showed a higher prevalence of PTSD attributed primarily to the nature of their job [8-10]. Also, as expected, patients affected with COVID-19 are more susceptible to PTSD [5,11,12].

The chief purpose of the current study originated from a logic inquiry of what would be the magnitude of PTSD in HCWs, specifically in COVID-19 positive HCWs workers. We hypothesized the risk of PTSD is higher among HCWs diagnosed with COVID-19 for two main reasons: first is that being a health worker dealing with patients during epidemics or pandemics carry a high risk of developing PTSD, as they are facing critical cases and witnessing mortalities; second is being COVID-19 positive potentially increases the risk of PTSD.

Out of all interviewed HCWs who had been survived COVID-19, 14.9% were classified as being positive for PTSD. Li et al. (2021) showed that the prevalence of PTSD among HCWs in general who were working during the COVID-19 pandemic was 21.5% (95% CI, 10.5%-34.9%) [13]. In this respect, Johnson et al. (2020) pointed that working directly with patients had a significantly increased risk of PTSD due to worries about job and economy, burnout, and health anxiety [14]. Kang et al. (2020) added that the fear of the HCWs about being responsible for transferring the infection to their relatives was a significant predictor of PTSD among HCWs [15].​​​​​​​

Surviving COVID-19 is an independent risk factor for developing PTSD; Janiri et al. (2021) pointed to the individuals who have had severe COVID-19 and feared imminent death from the disease, had family members and patients dead as a result of the disease, and witnessed COVID-19 patients’ life-threatening situations were more prone to have PTSD [12].​​​​​​​ In an interesting study, Liang et al. (2020) explained the bi‐phasic stress response model describing PTSD that could describe the vicious circle of PTSD with acquiring communicable diseases such as COVID-19 in which, despite the acute stress enhance immune response, chronic stress may suppress the immune response with increased susceptibility to infections [16].​​​​​​​ 

Other risk factors that significantly potentiated the risk of PTSD in our group included being affected with chronic disease. The distress of chronic disease patients towards COVID-19 partly refers to their compromised general health caused by pre-existing conditions, which renders them susceptible to the infection and a higher risk of mortality. Another practical aspect of concern for patients with pre-existing conditions would be postponement and inaccessibility to medical services and treatment as a result of the COVID-19 pandemic. For example, as rapidly growing numbers of COVID-19 patients were utilizing hospital and medical resources, primary, secondary, and tertiary prevention of other diseases may have unintentionally been affected [17].​​​​​​​

In our study, diabetic patients showed a significantly higher susceptibility to PTSD in the investigated HCWs. In this regard; Joensen et al. (2020) signified that diabetic patients have specific worries to COVID‐19, being characterized as a risk group due to diabetes and not being able to manage diabetes if infected; also, feeling isolated and lonely, and having changed diabetes behaviors such as checking of blood glucose levels and taking their medications, were associated with being more worried about COVID‐19 and diabetes [18].​​​​​​​

The HCWs affected with obesity who survived COVID-19 showed a significantly higher tendency to get PTSD and it is assumed that HCWs are expected to be knowledgeable about the facts that COVID-19 infection is not a good sign in obese patients, as it can cause more severe symptoms and complications; also patients affected with obesity may have difficulties during intubation, transportation, and receiving nursing care such as positioning, bathing, etc. [19].​​​​​​​ Moreover, HCWs are aware that obesity is a significant risk factor for severe COVID-19 complications, critical illness, and death [20].​​​​​​​

The regression model showed that being isolated in a hospital during COVID-19 infection is a significant predictor for developing PTSD. The possible causes could be related to: first, the additional adverse effects on brain function and mental health in patients with severe COVID-19 that necessitated hospital isolation [21];​​​ second, corticosteroids may induce affective psychosis, and hydroxychloroquine use has been related to agitation, emotional lability, and irritability [22]; third, the psychological pressure on COVID-19 patients due to isolation treatment may be far greater than that of medical workers and general residents [23].​​​​​​​

Another predictor for PTSD in the HCWs who survived COVID-19 was the nature of the job, which necessitates contact with positive cases. Similar findings were reported in China, where they found that the psychological burden was much higher in frontline HCWs directly engaged in the diagnosis, treatment, and care for patients with COVID-19 [9].​​​​​​​

Limitations

Despite several important results of this study, certain limitations should be considered. First, our study was conducted using a cross-sectional design, which cannot provide strong evidence for causality. Thus, a longitudinal design should be used in future research. Second, this study reflects only one city, thus generalization would be partly inappropriate, but it does draw attention to this issue. Future nationwide studies yielding conclusive numbers and an accurate estimate of prevalence are needed.

## Conclusions

The prevalence of PTSD in HCWs who survived COVID-19 infection came within the range of that in HCWs who were dealing with cases of COVID-19 without getting infected. The prevalence was significantly higher in HCWs who had chronic diseases, especially diabetics and obesity, HCWs whose job necessitates contact with positive cases, and HCWs who were isolated in hospitals while infected. 

Efforts should be made to alleviate stress in HCWs during their work in pandemics; especially those in the frontline, dealing directly with cases of COVID-19 and those who are suffering from chronic diseases. We recommend the initiation and implementation of tailored preventive mental health programs directed toward the well-being of HCWs during the crisis.
